# NK-, NKT- and CD8-Derived IFNγ Drives Myeloid Cell Activation and Erythrophagocytosis, Resulting in Trypanosomosis-Associated Acute Anemia

**DOI:** 10.1371/journal.ppat.1004964

**Published:** 2015-06-12

**Authors:** Jennifer Cnops, Carl De Trez, Benoit Stijlemans, Jiri Keirsse, Florence Kauffmann, Mark Barkhuizen, Roanne Keeton, Louis Boon, Frank Brombacher, Stefan Magez

**Affiliations:** 1 Laboratory of Cellular and Molecular Immunology, Vrije Universiteit Brussel, Brussels, Belgium; 2 Department of Structural Biology, Vlaams Instituut voor Biotechnologie (VIB), Brussels, Belgium; 3 Department of Myeloid Cell Immunology, VIB, Brussels, Belgium; 4 International Centre for Genetic Engineering and Biotechnology, Cape Town Component, Cape Town, South Africa; 5 Faculty of Health Sciences (IDM, Division Immunology), University of Cape Town, Cape Town, South Africa; 6 Bioceros, Utrecht, The Netherlands; National Institute of Health, UNITED STATES

## Abstract

African trypanosomes are the causative agents of Human African Trypanosomosis (HAT/Sleeping Sickness) and Animal African Trypanosomosis (AAT/Nagana). A common hallmark of African trypanosome infections is inflammation. In murine trypanosomosis, the onset of inflammation occurs rapidly after infection and is manifested by an influx of myeloid cells in both liver and spleen, accompanied by a burst of serum pro-inflammatory cytokines. Within 48 hours after reaching peak parasitemia, acute anemia develops and the percentage of red blood cells drops by 50%. Using a newly developed in vivo erythrophagocytosis assay, we recently demonstrated that activated cells of the myeloid phagocytic system display enhanced erythrophagocytosis causing acute anemia. Here, we aimed to elucidate the mechanism and immune pathway behind this phenomenon in a murine model for trypanosomosis. Results indicate that IFNγ plays a crucial role in the recruitment and activation of erythrophagocytic myeloid cells, as mice lacking the IFNγ receptor were partially protected against trypanosomosis-associated inflammation and acute anemia. NK and NKT cells were the earliest source of IFNγ during *T*. *b*. *brucei* infection. Later in infection, CD8+ and to a lesser extent CD4+ T cells become the main IFNγ producers. Cell depletion and transfer experiments indicated that during infection the absence of NK, NKT and CD8+ T cells, but not CD4+ T cells, resulted in a reduced anemic phenotype similar to trypanosome infected IFNγR-/- mice. Collectively, this study shows that NK, NKT and CD8+ T cell-derived IFNγ is a critical mediator in trypanosomosis-associated pathology, driving enhanced erythrophagocytosis by myeloid phagocytic cells and the induction of acute inflammation-associated anemia.

## Author Summary

African trypanosomes are the causative agents of Human and Animal African Trypanosomosis, impairing economic development and causing death throughout the African continent. Anemia and inflammation are hallmark features of virtually every type of trypanosome infection. During experimental murine trypanosomosis, early inflammation causes enhanced red blood cell phagocytosis by cells of the myeloid phagocytic system, leading to severe anemia within 48 hours past peak parasitemia. Here, we identify the pro-inflammatory cytokine IFNγ as the main driver of the early inflammatory reaction and enhanced red blood cell phagocytosis. This IFNγ is derived consecutively by NK, NKT and CD8+ T cells, hence these cells all play a crucial role in the induction of inflammation and anemia.

## Introduction

African trypanosomes cause a wide range of disease phenotypes, but a common hallmark of the infection is inflammation. Early during the course of infection, myeloid cells get activated by released parasite components such as soluble variant surface glycoproteins (sVSG) and DNA [1–7]. This gives rise to a type 1 cytokine storm which is critical for resistance [6,8–11], but is also associated with pathology development [12–16]. Indeed, coinciding with the acute inflammatory reaction, acute anemia develops, as witnessed by a 50% reduction in circulating red blood cells (RBC) within two days following peak parasitemia. After a short recovery phase, a subsequent gradually increasing loss of RBCs occurs during the chronic infection stage [13,17]. Anemia development is independent of antibodies [18] and the height of the parasitemia peak [17], and the acute nature of this phenomenon implies a consumptive etiology. Using a newly developed *in vivo* erythrophagocytosis assay, we have recently shown that acute anemia during Trypanosome infection is caused by enhanced RBC phagocytosis by activated cells of the myeloid phagocytic system, in combination with a decrease in RBC membrane stability [19]. More specifically, during the acute phase of *T*. *b*. *brucei* infection, activated liver neutrophils and monocytic cells (comprising monocytes and monocyte-derived macrophages) as well as activated spleen resident macrophages display enhanced erythrophagocytosis. This, in combination with the decreased RBC membrane stability, leads to disproportionate amount of RBC phagocytosis and hence acute anemia [19]. It is suggested that cells of the myeloid phagocytic system are ‘over’-activated by the type 1 induced inflammation early in infection, however the exact mechanism and pathway by which this occurs is unknown.

Previous studies on African trypanosome infections have established that IFNγ is required to prime macrophages in order to become fully activated and induce an efficient type 1 response [2,3,6,20]. This indicates that IFNγ production occurs very early in infection, even before macrophage activation. Although no direct evidence was provided, others have implied CD8 T cells [21–24] and VSG-specific CD4 T cells [9] to be potential sources of IFNγ during African trypanosome infections. In addition, it was recently shown in murine *Toxoplasma gondii* infection that IFNγ can act directly on macrophages to provoke RBC uptake [25].

In this study we aimed to elucidate the mechanism(s) and immune pathway(s) responsible for the induction of acute anemia during African trypanosome infection.

Here, using the clonal laboratory-adapted *Trypanosoma brucei brucei (T*. *b*. *brucei)* strain, we show that mice lacking the IFNγ receptor suffer less from infection-associated inflammation and acute anemia. Moreover, we show for the first time that during experimental trypanosome infections NK and NKT cells are the earliest IFNγ producers, followed by CD8 and CD4 T cells, and that IFNγ plays a crucial role in the recruitment and activation of erythrophagocytic myeloid cells. In addition, the results indicate that the absence of NK, NKT and CD8 T cells, but not CD4 T cells, during the early stage of infection results in a reduced anemic phenotype similar to IFNγR-/- mice.

Collectively, this study shows that NK-, NKT- and CD8-derived IFNγ is crucial for enhanced erythrophagocytosis by myeloid phagocytic cells and consequently for the induction of acute inflammation-associated anemia.

## Materials and Methods

### Mice

6–8 week old female C57BL/6 mice were purchased from Janvier. C57BL/6, IFNγ-/- and IFNγR-/- mice were obtained through Dr. B. Ryffel (CNRS, Orleans, France). The interferon-gamma reporter with endogenous polyA transcript (GREAT) mice were purchased from The Jackson Laboratory. These mice were housed in individual ventilated cages at the Vrije Universiteit Brussel.

C57BL/6 CD4-/-, CD8-/- and C57BL/6 nu/nu mice were a kind gift from Dr. H. Mossmann (MaxPlanck Institute, Freiburg, Germany). These mice were housed in individual ventilated cages and maintained in SPF barrier facilities at the University of Cape Town.

### Ethics statement

All experiments complied with the ECPVA guidelines and were approved by the ETHICAL COMMITTEE for ANIMAL EXPERIMENTS (ECAE) at the Vrije Universiteit Brussel (protocol #14-220-23 and #12-220-2) and the University of Cape Town, South Africa # 97/001 and 005/041.

### Parasites, infections and red blood cell counts

Mice were infected by intraperitoneal (i.p.) injection of 5000 pleomorphic *Trypanosoma brucei brucei* AnTat1.1E parasites, which were a kind gift from N. Van Meirvenne (Institute for Tropical Medicine, Belgium). RBC counts were determined via a hematocytometer at two to four day intervals on 2,5μl blood sample collected from the tail vein and diluted 1/200 in PBS. Anemia was expressed as the percentage of reduction in RBC counts compared to non-infected animals.

### Cell depletions and neutralization experiments

For depletion of CD8 T cells, mice received the first i.p. injection of 500μg anti-CD8 Ly2 rat-anti-mouse monoclonal antibody 24 hours prior to infection. Subsequently, mice received a dose of 100 μg 2 day intervals post infection. NK and NKT cells were depleted with the anti-NK1.1 PK136 rat-anti-mouse monoclonal antibody. 250μg was given four and one day prior to infection. A dose of 300μg was given at 2–3 day intervals post infection. Depletion efficiency of CD8 T cells and NK(T) cells from both spleen and liver was assessed by flow cytometry.

For neutralization of IFNγ, wild type mice were treated with 500μg neutralizing IFNγ antibody (clone XMG1.2, Bioceros) at two-day intervals, starting at day 1 post infection. Control mice were treated with corresponding volumes of PBS.

### Cell and serum isolation and cell culture

Blood was harvested from CO_2_ euthanized mice by cardiac puncture and centrifuged at 10000rpm for 10 min. Serum was harvested and stored at -20°C.

Spleen and liver were harvested from CO_2_ euthanized mice. For myeloid cell analysis livers were perfused with cold PBS prior to harvesting. Consecutively, these livers were minced in 10 ml digestive medium (0.05% collagenase type A in Hanks' Balanced Salt Solution (HBSS); Invitrogen) and incubated at 37°C for 30 min. The digested tissue was then homogenized (GentleMacs) and filtered (40 μm pore filter). For analysis of lymphocytes, livers were homogenized and filtered (40 μm) and restricted to a 33% Percol in PBS gradient (1800 rpm, 12 min, room temperature). Spleen cells were obtained by homogenizing the organs in 10 ml RPMI medium containing 5% fetal calf serum (FCS) and filtered (40 μm pore filter). Next, liver and spleen cell suspension were centrifuged (1400 rpm, 7 min, 4°C) and the pellet treated with RBC lysis buffer (0.15 M NH_4_Cl, 1.0 mM KHCO_3_, 0.1 mM Na_2_-EDTA). Subsequently, the cells were resuspended in CM–medium (RPMI medium, 5%FCS, 1% L-glutamine, 1% Penicillin-Streptomycin) or RPMI 5% FCS or plain RPMI for cell culture flow cytometry or cell isolation respectively. For cell culture 3 10^5^ cells were put in flat bottom 96-well plates and incubated at 37°C and 5% CO_2_. Cell culture supernatant was harvested after 24 or 48 hours and stored at -20°C.

### Adoptive transfer experiments

CD8-/- mice were reconstituted with splenocytes from naïve C57BL/6 donor mice. CD8 T cells were purified *via* negative selection using the EasySep Mouse CD8+ T Cell Isolation kit according to the manufacturers protocol (StemCell Technologies). Obtained cell suspensions were between 80 and 90% pure. CD8 T cells were carboxyfluorescein succinimidyl ester (CFSE) labeled allowing retracement of transferred cells in acceptor mice (S1). Briefly, CD8 T cells were put at a concentration of 10^7^ cells per ml and incubated with 5μM CFSE for 15 min at 37°C 5% CO_2_. Subsequently, labeled cells were incubated for 15 minutes with 10 ml PBS 1%BSA at 37°C, 5% CO_2_ and washed twice with the same medium at 2000 rpm, 7 minutes. Between 5x10^6^ and 1x10^7^ CD8 T cells were injected i.v. into acceptor mice 24 hours prior to infection and four days post infection.

C57BL/6 nu/nu mice were reconstituted with splenocytes from naïve C57BL/6 donor mice. CD8+ and CD4+ T-cell purification was performed using the antibody cocktail and density gradient method (Stem Cell Technologies) according to the manufacturers’ protocol. Obtained cell suspensions were 95% pure. 3 10^7^ cells were injected i.p. into acceptor mice 24 hours prior to infection. CD4+ T-cell reconstituted mice were given an additional injection of 500 μg anti-CD8 mAb, 2 hours after cell reconstitution.

### Red blood cell labeling and *in vivo* erythrophagocytosis assay

Blood was harvested from CO_2_ euthanized mice by cardiac puncture using 50μl 1000 U/ml heparin. RBCs were counted and 10^9^ RBCs were washed twice with 15 ml PBS, 2000 rpm, 7 minutes. Next, RBCs were labeled with 2 μl pHrodo Red succinimidyl ester (pHrodo Red, Life Technologies) in a final volume of 1 ml PBS for 60 minutes at 37°C 5% CO_2_. Subsequently, labeled RBCs were incubated for 15 minutes with 10 ml RPMI/5% FCS at 37°C and washed twice with the same medium, 2000 rpm, 7 minutes. Labeled RBCs were resuspended in RPMI. As negative control, and for the determination of the background signal, RBCs were treated in the same manner without addition of the pHrodo dye. 7–8 week old female C57BL/6 non-infected or *T*. *brucei* infected (day 6 p.i.) mice were injected intravenously (i.v.) with 10^9^ pHrodo labeled or unlabeled RBCs in 200 μl RPMI. After 18 hours, mice were CO_2_ euthanized and spleen and livers were isolated and processed into single cell suspension as described above. Next, the cells were analyzed via flow cytometry as described further.

### *In vitro* erythrophagocytosis essay

Spleen cells were isolated as described and resuspended in ME—medium (RPMI medium, 5% FCS, 1% L-glutamine and non essential amino acids, 1% Penicillin-Streptomycin) at a concentration of 4 105 cells per 200 μl. Red blood cells were isolated and labeled as described and 2 107 labeled or unlabeled RBCs were put in co-culture with 4 105 cells in polypropylene tubes (BD Biosciences). Co-cultures were incubated overnight at 37°C and 5% CO2 with or without IFNγ stimulation (100U/ml). After overnight culture, cells were submitted to flow-cytometrical analysis.

### Flow cytometry

Cells were washed with FACS medium (5% FCS in RPMI) and non-specific binding sites were blocked by incubating 20 minutes at 4°C with an Fc-blocking antibody (anti-CD16/32, clone 2.4G2). Next, cell suspensions were stained with fluorescent conjugated antibodies for 30 minutes at 4°C. Fluorescent antibodies: CD11b PE-Cy7 clone M1/70, F4/80 FITC clone C1:A3-A, Ly6C APC clone AL-21, Ly6G PerCP-Cy5.5 clone 1A8, CD45 APC-Cy7 clone 30-F11, CD4 BV421 clone GK1.5, CD8 BV510 clone 53–67, NK11 PE clone PK136 (BD Biosciences), CD64 Pe clone X54-5/7.1. (BioLegend), CCR2 Pe clone 475301, MerTK Pe clone 108928 (R&D systems), Ly6B clone 7/4 (AbD Serotec)., TCRb APC clone H57-597, CD49b Pecy7 clone DX5, NKp46 PE clone 29A1.4 (eBioscience). Following washing with FACS buffer they were analyzed on a FACS Canto II flow cytometer (BD Biosciences) and data was processed using FlowJo software (Tree Star Inc.).

### Cytokine analysis

Concentrations of IL15/IL15R, IL12p70 and TNFα (R&D Systems) as well as IFNγ (Pharmingen) in serum and cell supernatant were determined by ELISA according to the manufacturers’ protocol.

### Statistical analysis

Statistical analysis was performed using Student-test and GraphPad Prism software (GraphPad 6, San Diego, CA). Values are expressed as mean ± standard deviation (SD) unless stated otherwise. Values of p≤ 0.05 are considered to be statistically significant.

## Results

### IFNγ-activated and recruited myeloid cells induce inflammation-associated acute anemia

**Fig 1:**
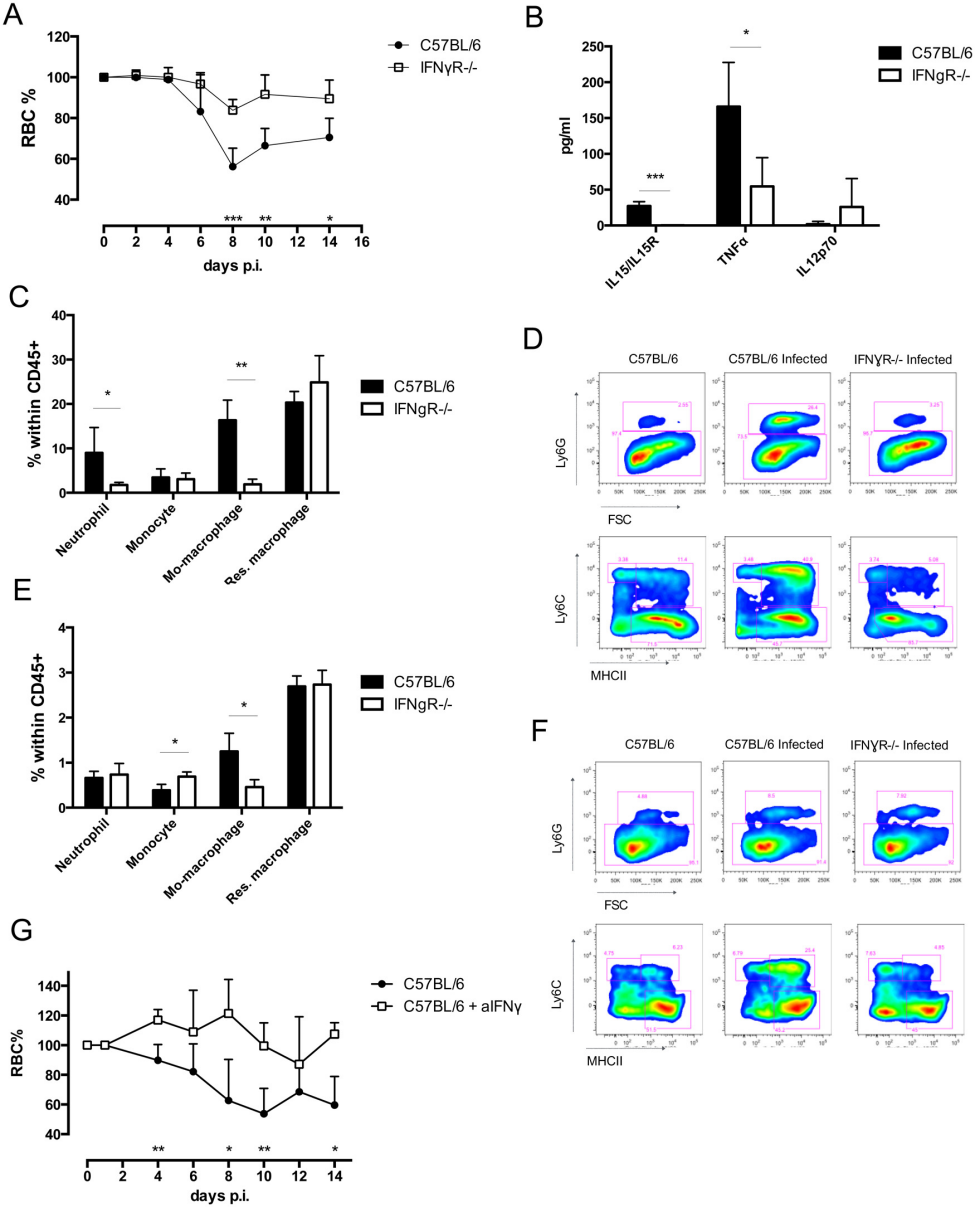
Analysis of cell composition and serum cytokines in IFNγR-/- mice. A) Anemia profile of *T*. *b*. *brucei* infected C57BL/6 mice and IFNγR-/- mice. RBC counts were normalized individually compared to naïve mice. Values are presented as mean ± SD of 5 mice per group. At each time point the difference in RBC% between infected C57BL/6 mice and knock out mice was evaluated. B) Serum concentration of IL15/IL15R complex, TNFα, and IL12p70. Values represent mean ± SD of 4 mice per group. C) Composition of myeloid cells within CD45+ liver cells. Values represent mean ± SD of 4 mice per group. D) Facs plots of myeloid cells in liver. E) Composition of myeloid cells within CD45+ spleen cells. Values represent mean ± SD of 4 mice per group. F) FACS plots of myeloid cells in spleen. One representative out of two three similar experiments is shown. G) Anemia profile of *T*. *b*. *brucei* infected anti-IFNγ treated C57BL/6 mice and infected control PBS-infused C57BL/6 mice. RBC counts were normalized individually compared to naïve mice. At each time point the difference in RBC% between infected C57BL/6 mice and IFNγ infused mice was evaluated. Values are presented as mean ± SD of 4 mice per group *: p-value < 0.05; **: p-value < 0.01; ***: p-value < 0.005 and if nothing is mentioned the differences were not significant.

Trypanosome infections are characterized by multiple parasitemia waves and a survival of approximately 30–35 days [8,12]. The peak of parasitemia occurs at day 5–6 post infection, followed by acute anemia development [17]. Previous research on African trypanosome infections has established an important role for IFNγ during the onset of infection. Indeed, IFNγ is crucial for macrophage activation and optimal initiation of the type 1 immune response associated with resistance to infection [6,11]. Coinciding with the peak of parasitemia and induction of anemia (day 6), a burst in serum pro-inflammatory cytokines is observed [14,17]. To investigate the role of IFNγ during *T*. *b*. *brucei* infection-associated pathology, IFNγR-/- mice were infected and anemia was monitored. Infected IFNγR-/- mice suffered much less from acute anemia compared to infected C57BL/6 mice (Fig 1A). Coinciding in IFNγR-/- mice, reduced amounts of pro-inflammatory cytokines IL-15 and TNFα were observed at the time of peak parasitemia (day 6) (Fig 1B).

To investigate the contribution of IFNγ in the alteration of the myeloid cell composition a detailed investigation (gating strategy S1A and S1B Fig) of liver and spleen cell composition was performed. The liver of IFNγR-/- mice showed a reduced influx of neutrophils (defined as CD11b+, Ly6G+) and monocyte-derived macrophages (defined as CD11b+, Ly6C+, MHCII+) at day 4 post infection (Fig 1C and S1C Fig). No changes were observed in monocytes (defined as CD11b+, Ly6C+, MHCII-) and resident macrophages (defined as CD11b+, Ly6C-, MHCII+). Therefore, the composition of liver myeloid cells of infected IFNγR-/- mice closely resembled that of naïve C57BL/6 mice (Fig 1D). Of note, there was no difference in myeloid cell composition between naïve C57BL/6 and IFNγR-/- mice. Similarly, the myeloid cell composition in the spleen of infected IFNγR-/- mice showed a reduced influx of monocyte-derived macrophages, without differences in percentages of resident macrophages, when compared to infected C57BL/6 mice (Fig 1E and 1F, S1D Fig). In contrast to the situation in the liver, neutrophil influx was similar to wild type mice, and the proportion of monocytes within the CD45+ population increased in IFNγR-/- mice, when compared to wild type mice (Fig 1E and 1F, S1D Fig). Treatment of infected C57BL/6 mice with neutralizing IFNγ antibody resulted in maintenance of RBC during the acute stage of the infection, confirming the result described above in IFNγR-/- mice (Fig 1G).

**Fig 2:**
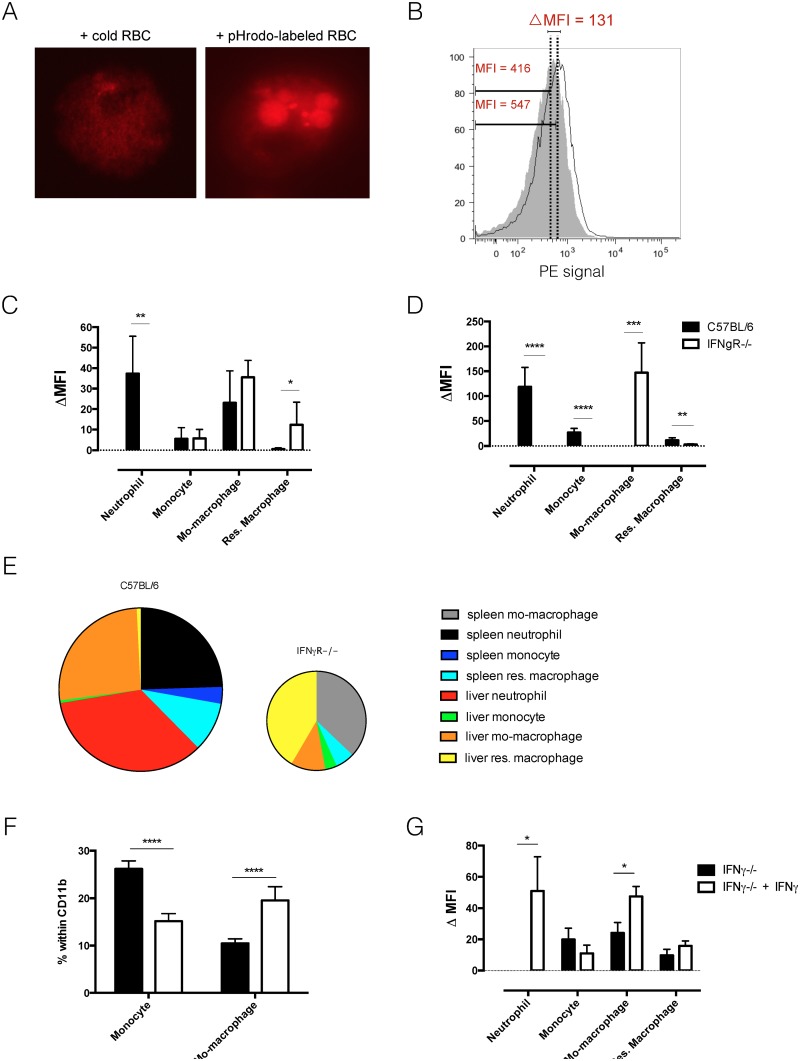
*In vivo* erythrophagocytosis assay and *ex vivo* RBC hemolysis experiment. A) *Ex vivo* fluorescent microscopy picture of erythrophagocytozing cell in the spleen of *T*. *b*. *brucei* infected (day 6 p.i.) mice. Left panel: co-culture of spleen cells with unlabeled RBC. Right panel: co-culture of spleen cells with pHrodo-labeled RBC. B) Histogram of PE signal of liver macrophages exposed *in vivo* to unlabeled RBC (grey tinted area) and pHrodo – labeled RBC (single black line). The delta MFI is the difference between the median fluorescent intensities of the two cell populations. D&E) *In vivo* erythrophagocytosis assay: delta median fluorescent intensity of myeloid cells in liver (C) and spleen (D) of *T*. *brucei* infected (day 6 p.i.) mice. Values represent mean ± SEM of 6 mice per group. E) Relative contribution to phagocytosis: cell numbers were multiplied by the deltaMFI observed for each subpopulation. The surface of the diagram of IFNγR-/- mice displays the total number of phagocytic cells and is calculated relatively to the surface of the diagram of C57BL/6 mice. F) *In vitro* stimulation of naïve monocytes derived from IFNγ-/- mice with IFNγ induces differentiation to monocyte-derived macrophages. Values represent mean +/- SD of 6 mice per group. G) *In vitro* erythrophagocytosis assay to determine the effect of IFNγ stimulation on naïve spleen cells from IFNγ-/- mice. Values represent mean ± SEM of 6 mice per group. One representative of 2 independent experiments is shown. *: p-value < 0.05; **: p-value < 0.01; ***: p-value < 0.005; ****: p-value < 0.0001 and if nothing is mentioned the differences were not significant.

To determine the phagocytozing capacity of these distinct myeloid cell subsets in IFNγR-/- mice, a newly developed *in vivo* pHrodo-based erythrophagocytosis assay was used [19]. In this assay, RBCs are labeled with the acid-sensitive dye pHrodo *ex vivo* prior to i.v. injection in recipient mice. Following lysosomal uptake of labeled RBCs, phagocytozing cells become fluorescent (Fig 2A). The change in fluorescent intensity is then monitored between cells that have taken up unlabeled RBC and labeled RBC and expressed as delta median fluorescent intensity (ΔMFI) (Fig 2B). Liver neutrophils, monocytic cells, as well as spleen resident macrophages showed an increase in erythrophagocytozing capacity upon *T*. *b*. *brucei* infection in C57BL/6 ([19] and Fig 2C). In striking contrast, the erythrophagocytozing capacity of neutrophils drastically dropped in IFNγR-/- mice (Fig 2C and 2E). The erythrophagocytozing capacity of monocytic cells in IFNγR-/- mice was equal to that of wild type mice (Fig 2C). However, as IFNγR-/- mice display a remarkably reduced influx of monocyte-derived macrophages in the liver (Fig 1C and S1C Fig), the contribution of these cells to anemia development in IFNγR-/- mice could be minor. In contrast to wild type mice, liver resident macrophages of IFNγR-/- mice showed significant erythrophagocytozing potential (Fig 2C and 2E). Again, this resembles the situation of naïve mice, in which liver resident macrophages are the main cells involved in RBC uptake. In the spleen of infected wild type mice, neutrophils and resident macrophages showed the highest erythrophagocytozing potential (Fig 2D and 2E), whereas in IFNγR-/- mice the erythrophagocytozing capacity of these cell populations dropped significantly (Fig 2D and 2E). Spleen monocytes, which were present in higher amounts in IFNγR-/- mice compared to wild type mice (Fig 1D and 1E, S1D Fig), also showed a reduced erythrophagocytozing potential (Fig 2D). However, the erythrophagocytozing potential of monocyte-derived macrophages of IFNγR-/- mice was enhanced compared to that of wild type mice (Fig 2D and 2E). Yet, taking into account the low abundance of the monocyte-derived macrophages of IFNγR-/- mice compared to wild type mice, their contribution to acute anemia induction may be minor.

Using the *in vitro* pHrodo-erythrophagocytosis assay [19] we investigated the direct effect of IFNγ signaling on myeloid cells. In this setup, cells from uninfected, IFNγ-/- mice were incubated with labeled RBC and unlabeled RBC (background) in the presence or absence of IFNγ. Cellular composition and erythrophagocytosis potential was analyzed. As shown in Fig 2F, addition of IFNγ to cells from IFNγ-/- mice led to a shift in monocyte and monocyte-derived macrophage percentage. As the percentage of monocytes decreased, the percentage of monocyte-derived macrophages increased (Fig 2F). This suggests that IFNγ signaling could directly induce the differentiation of monocytes into monocyte-derived macrophages. Other surface markers should however be investigated to determine if these cells are indeed monocyte-derived macrophages. Fig 2G shows that upon incubation of naïve cells with RBC, IFNγ stimulation directly induces an up-regulation of the erythrophagocytic potential of neutrophils and monocyte-derived macrophages. This result suggests that neutrophils and monocyte-derived macrophages can directly alter their erythrophagocytic potential upon IFNγ signaling. For neutrophils it seems that IFNγ is a crucial inducer of erythrophagocytosis, as the erythrophagocytic potential is completely absent in neutrophils from IFNγ-/- mice (Fig 2G) and IFNγR-/- mice. This effect has been described before [26]. In contrast, monocyte-derived macrophages are still able to phagocytoze RBC in the absence of IFNγ, suggesting that IFNγ is not crucial for erythrophagocytosis by monocyte-derived macrophages.

**Fig 3:**
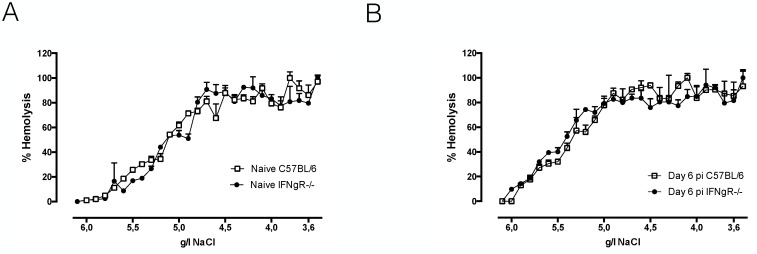
Hemolysis experiment: osmotic fragility profile of naïve (A) or infected (B) RBCs of day six infected C57BL/6 (white line) or IFNγR-/- (black line) mice. RBCs were incubated with decreasing concentrations of NaCl, resulting in hemolysis of RBCs. The percentage of hemolysis was plotted against the concentration of NaCl in the medium and the NaCl concentrations corresponding with 50% hemolysis were determined. As positive control RBCs were exposed to 100% distilled H_2_O and as negative control RBCs were exposed to 100% HBSS-solution. One representative out of two experiments is shown.

Recently, we showed that during *T*. *b*. *brucei* infection the lipid composition of circulating RBC is altered, which coincided with an increase in susceptibility to lysis [19]. This in turn can contribute to enhanced RBC uptake and acute anemia. To investigate the role of IFNγ in the alteration of RBC membrane fragility, we performed a hemolysis experiment using resistance to osmolarity as a read-out. As indicated in Fig 3A, no difference in RBC osmotic fragility of naïve wild type and IFNγR-/- mice was observed. Upon *T*. *b*. *brucei* infection the same increase in osmotic fragility was observed for RBC of both infected wild type and IFNγR-/- mice (Fig 3B).

In conclusion, IFNγ appears to be indispensable for induction of the classical type 1 inflammation, leading to myeloid cell activation and recruitment, and resulting in enhanced erythrophagocytosis and acute anemia during early *T*. *b*. *brucei* infection. In addition, infection-associated alteration of RBC membranes is independent of IFNγ and its contribution to acute inflammation-associated anemia development seems to be minor.

### NK and NKT cells are the earliest IFNγ producers during *T*. *b*. *brucei* infection

It is generally accepted that IFNγ plays a key role in early stages of infection and CD8 and CD4 T cells have been indirectly suggested to be the sources of IFNγ during *T*. *b*. *brucei* infection [9,21,22,24]. To investigate the cellular source of IFNγ, IFNγ reporter (GREAT) mice were infected with *T*. *b*. *brucei* and liver and spleen were analyzed for IFNγ production (gating strategy S2A).

**Fig 4:**
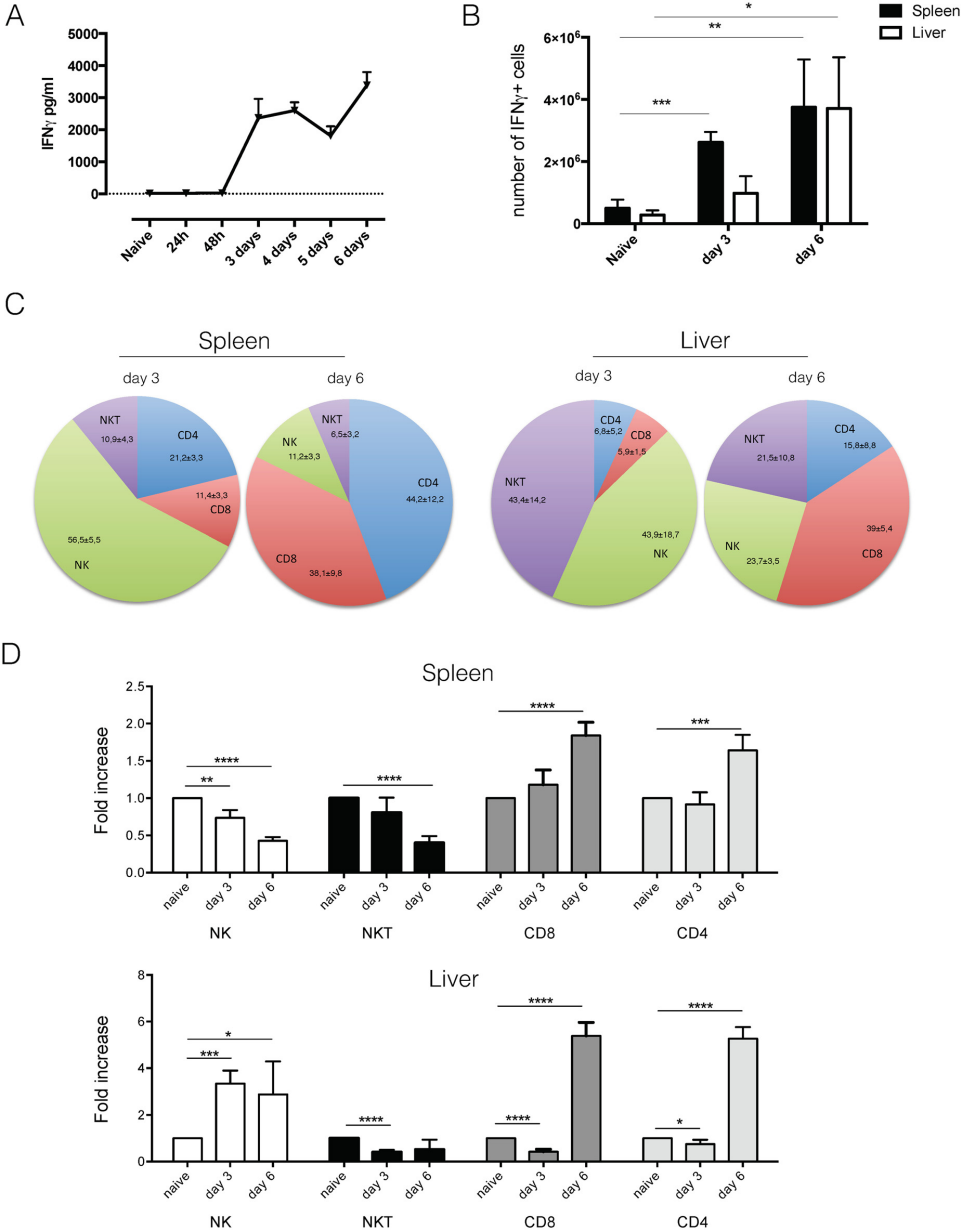
Analysis of IFNγ production using GREAT reporter mice. A) Serum cytokine concentration of IFNγ in infected C57BL/6 mice. Values represent mean ± SD of 4 mice. B) Number of IFNg producing cells in spleen and liver of *T*. *b*. *brucei* infected mice. Values represent mean ± SD of at least 3 mice. C) Composition of spleen and liver cells within IFNg producing cells. Numbers represent mean ± SD of at least 3 mice. D) Fold increase in number of NK, NKT, CD8 and CD4 T cells in liver and spleen following *T*. *b*. *brucei* infection. Values represent mean ± SD of 4 C57BL/6 mice. One representative out of two experiments is shown.*: p-value < 0.05; **: p-value < 0.01; ***: p-value < 0.005; ****: p-value < 0.0001 and if nothing is mentioned the differences were not significant.

As previously shown [14,17], systemic IFNγ was present in quantifiable amounts at day 3 post infection (Fig 4A). This corresponded to approximately 2.6 x 10^6^ IFNγ producing spleen cells and 0.9 x 10^6^ IFNγ producing cells in the liver (Fig 4B). At the time of anemia induction at day 6 post infection, both spleen and liver had equal amounts of IFNγ producing cells (approximately 3.7 x 10^6^) (Fig 4B). IFNγ production in GREAT mice was confirmed on the protein level in serum and spleen cell culture (S2B). Further detailed investigation into the cellular source of IFNγ showed that at day 3 post infection, NK cells were the dominant IFNγ producing population in the spleen, while in the liver both NK and NKT cells were the principal IFNγ producing cell populations (Fig 4C). By day 6 post infection, a shift occurred and CD4 and CD8 T cells were the majority of IFNγ producing cells in the spleen (Fig 4C). In the liver, the population of IFNγ producing NK and NKT cells also contracted, but to a lesser extent as in the spleen, and CD8 T cells become the dominant IFNγ producing cell population (Fig 4C). Upon investigation of the lymphocyte population dynamics in the spleen, a reduction in the amount of NK and NKT cells was observed by day 6 while an almost two-fold increase in CD8 T cells and CD4 T cells was observed (Fig 4D). In the liver an enormous expansion of NK cells occurred by day 3 post infection, while the NKT cell population quickly contracted (Fig 4D). CD8 T cells and CD4 T cells expanded approximately five-fold by day 6 post infection.

**Fig 5:**
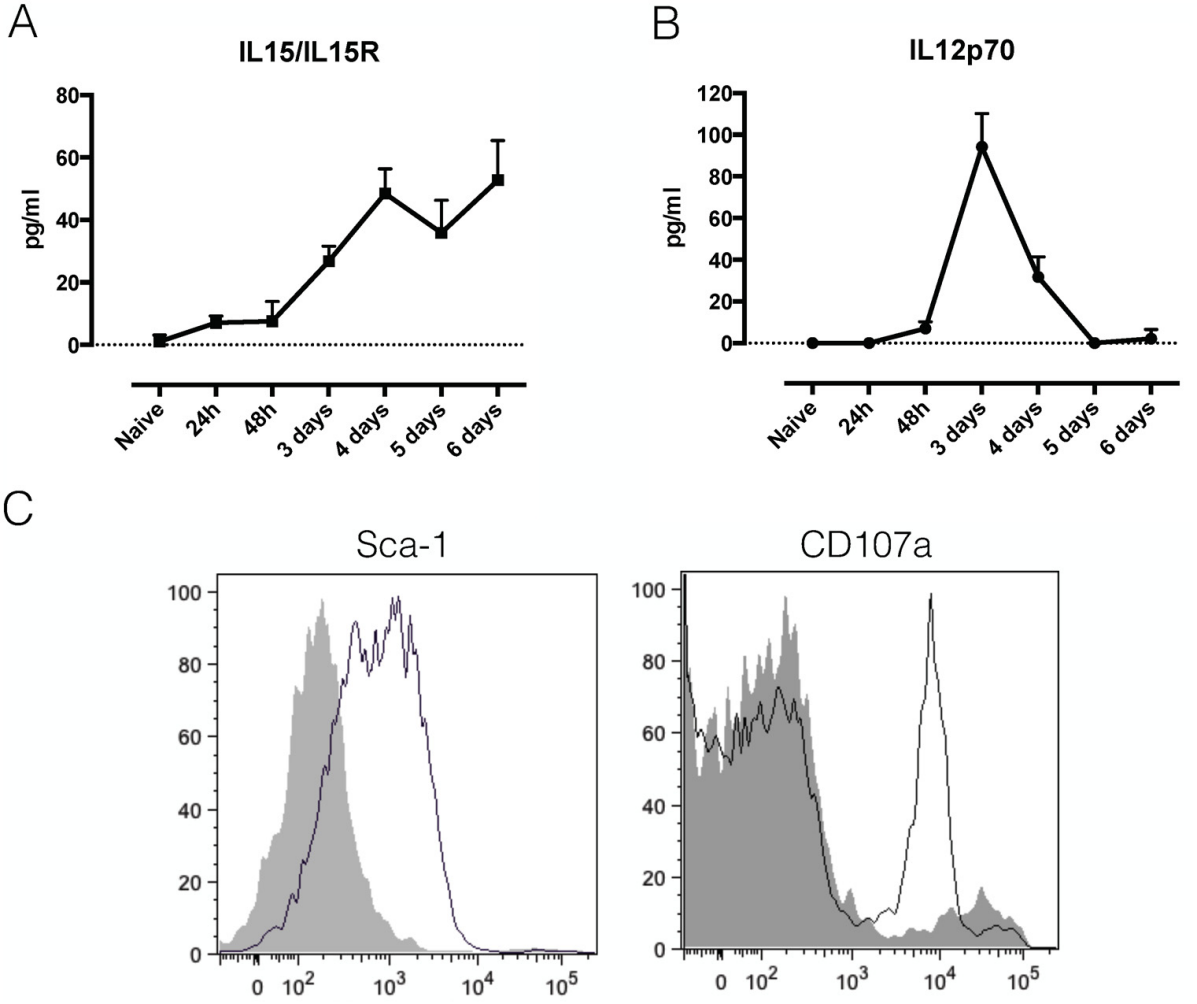
NK and NKT activation. A) Serum cytokine concentration of the IL15/IL15R complex in infected C57BL/6 mice. Values represent mean ± SD of 4 mice. B) Serum cytokine concentration of IL12p70 in infected C57BL/6 mice. Values represent mean ± SD of 4 mice. C) Histograms showing the expression of Sca-1 and CD107a on liver IFNγ-producing NK cells of naïve (grey tinted line) versus infected (simple black line) GREAT reporter mice. One representative out of two experiments is shown.

As this is the first time that NK and NKT cells have been implicated in *T*. *b*. *brucei* infection, we investigated possible activating mechanisms. For both NK and NKT cells, mechanisms of selective and non-selective activation have been described [27–29]. Investigation of serum cytokines indicated an early appearance of IL-12 and IL-15 (Fig 5A and 5B). While IL-15 generally drives NK cell proliferation and IFNγ production [30], IL-12 has been implicated in non-selective NKT cell activation [29]. Recent research into NK cell activation pathways have identified Stem cell antigen 1 (Sca-1) as a novel marker of non-selective NK cell activation. Investigation of surface markers of IFNγ producing NK cells showed that Sca-1, in addition to CD107a, is up regulated upon *T*. *b*. *brucei* infection (Fig 5C), indicating that they might be non-specifically activated.

Taken together, this data shows that both liver and spleen are important sources of IFNγ during *T*. *b*. *brucei* infection, and that NK and NKT cells are the earliest activated cells and IFNγ producers. Only by day 6 post infection, do CD8 and CD4 T cells become the dominant IFNγ sources.

### NK, NKT and CD8 T cells are essential for the induction of the acute anemic phenotype

**Fig 6:**
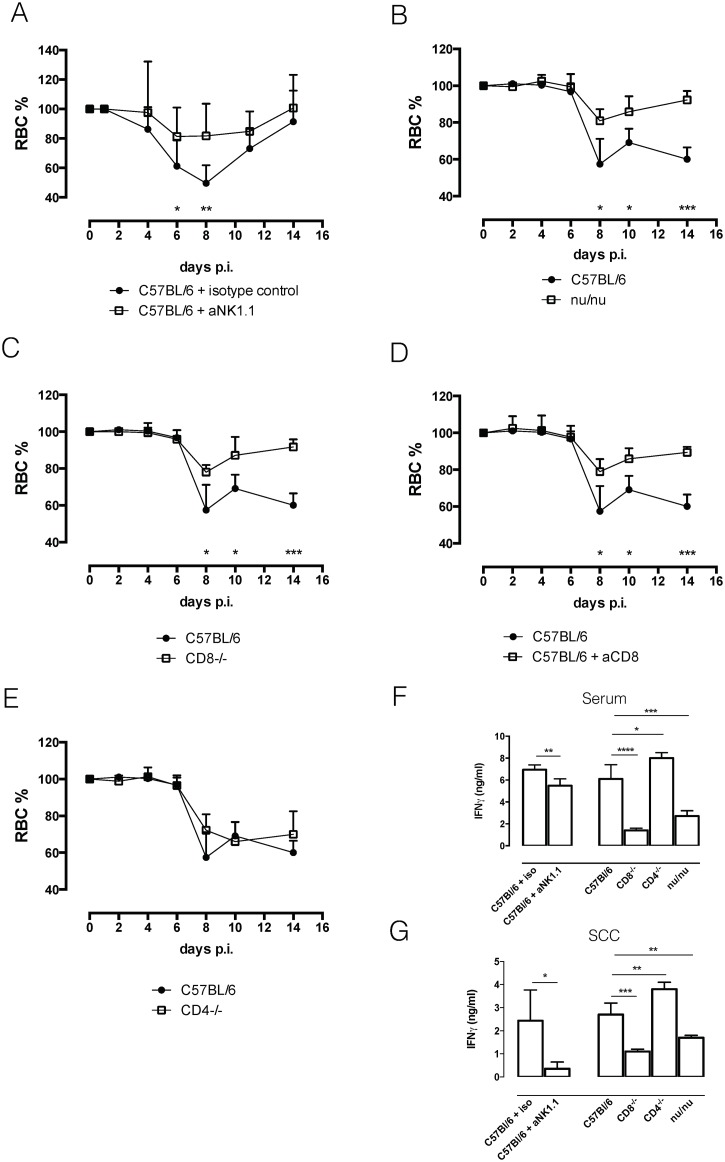
Anemia profiles and IFNγ production. A) Anemia profile of anti-NK1.1 infused mice and isotype-control treated mice. Values represent mean ± SD of 8 mice per group. B) Anemia profile of C57BL/6 and nu/nu mice. Values represent mean ± SD of 4 mice per group. C) Anemia profile of C57BL/6 and CD8-/- mice. Values represent mean ± SD of 4 mice per group. D) Anemia profile of C57BL/6 and anti-CD8 infused mice. Values represent mean ± SD of 4 mice per group. E) Anemia profile of C57Bl/6 and CD4-/- mice. Values represent mean ± SD of 4 mice per group. F) Serum IFNγ concentration. Values represent mean ± SD of at least 4 mice per group. G) IFNγ concentration in spleen cell culture supernatant (SCC). Values represent mean ± SD of at least 4 mice per group. One representative out of two experiments is shown. At each time point the difference in RBC% between infected C57BL/6 mice and knock out mice was evaluated. *: p-value < 0.05; **: p-value < 0.01; ***: p-value < 0.005; ****: p-value < 0.0001 and if nothing is mentioned the differences were not significant.

As IFNγR-/- mice did not suffer as much from the acute anemia observed in wild type C57BL/6 mice, we examined the contribution of each IFNγ producing cell subset to acute anemia induction and systemic IFNγ levels. As results from the previous section indicated that NK and NKT cells were the earliest IFNγ producing cells during *T*. *b*. *b*. infection, we infected C57BL/6 mice depleted of NK1.1+ cells (S3A). In the absence of both NK and NKT cells, *T*. *b*. *brucei* infected anti-NK1.1 treated mice suffered less from acute anemia compared to control mice (Fig 6A). This reduced anemic phenotype coincided with reduced levels of IFNγ in serum and spleen cell cultures (Fig 6F and 6G). By day 6 post infection, CD8 and CD4 T cells seemed to become the dominant IFNγ producing cells. Infection of nu/nu mice, lacking both CD4 and CD8 T cells, resulted in a diminished anemia phenotype compared to C57BL/6 mice (Fig 6B), and coincided with reduced levels of IFNγ in serum and spleen cell culture (Fig 6F and 6G). To specify whether CD8 and CD4 T cells are equally important for acute anemia induction, anti-CD8-treated mice, CD8-/- mice and CD4-/- mice were infected with *T*. *b*. *brucei*. Both CD8-/- mice and anti-CD8 treated mice showed reduced anemia compared to wild type C57BL/6 mice (Fig 6C and 6D), coinciding with reduced levels of IFNγ in serum and spleen (Fig 6F and 6G). In contrast, CD4-/- mice presented a similar anemic phenotype as wild type mice (Fig 6E), and serum and spleen cell culture IFNγ levels were similar between CD4-/- and C57BL/6 mice (Fig 6F and 6G).

**Fig 7:**
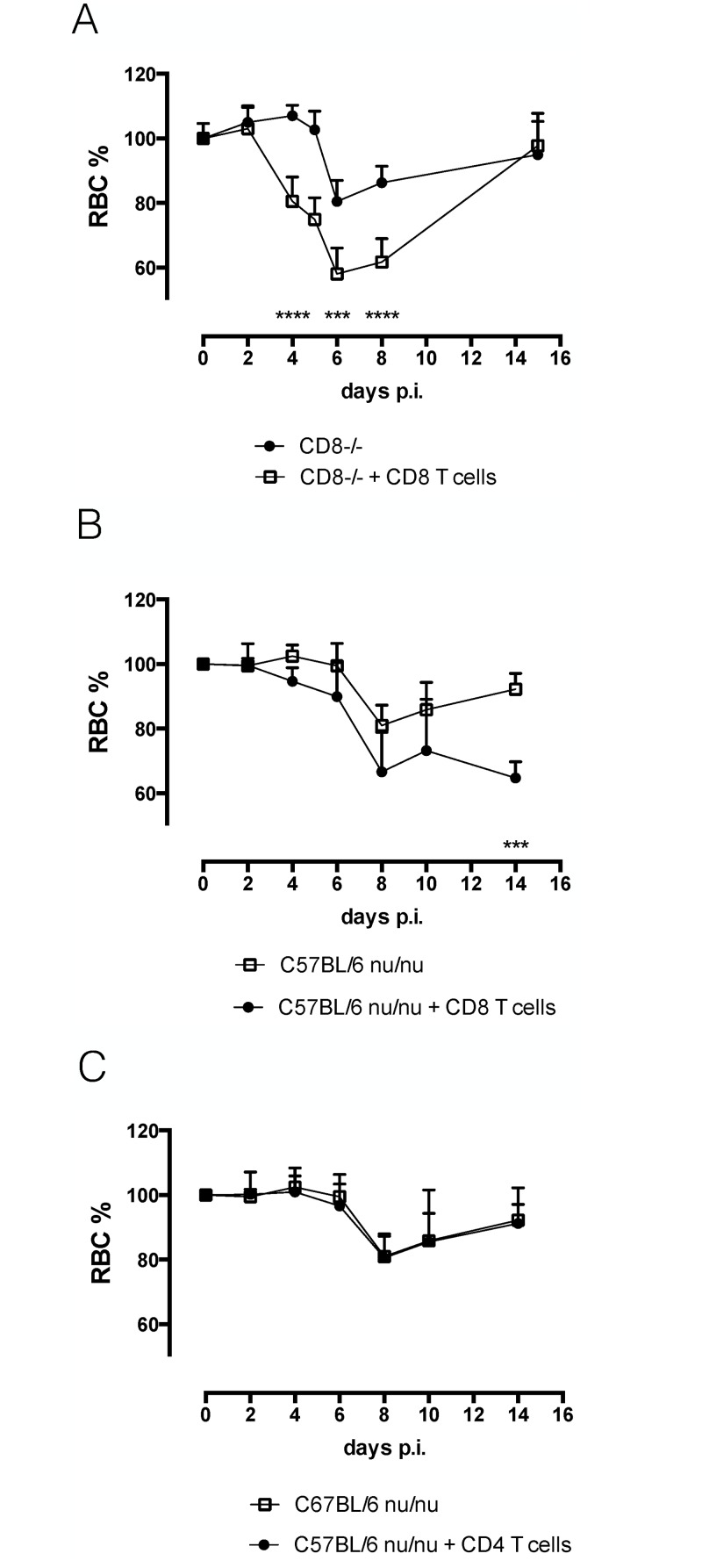
Reconstitution experiments. A) Anemia profile of CD8-/- mice and CD8 T cell reconstituted CD8-/- mice. Values represent mean ± SD of at 6 mice per group. One representative out of two experiments is shown. B) Anemia profile of nu/nu mice and CD8 T cell reconstituted nu/nu mice. Values represent mean ± SD of 4 mice per group. C) Anemia profile of nu/nu mice and CD4 T cell reconstituted nu/nu mice. Values represent mean ± SD of 4 mice per group. One representative out of two experiments is shown. At each time point the difference in RBC% between infected C57BL/6 mice and knock out mice was evaluated. ***: p-value < 0.005; ****: p-value < 0.0001 and if nothing is mentioned the differences were not significant.

To confirm the role of IFNγ-producing CD8 T cells in the induction of acute anemia, isolated C57BL/6 CD8 T cells were adoptively transferred in CD8-/- mice and C57BL/6 nu/nu mice prior to infection (Annex S3 Fig). Adoptive transfer of CD8 T cells in these knock out mice resulted in the induction of anemia in these mice (Fig 7A and 7B). As a negative control, C57BL/6 nu/nu mice were reconstituted with CD4 T cells (Fig 7C), which did not reverse the reduced anemic phenotype.

In conclusion, together, these results demonstrated a crucial role for NK1.1+ and CD8 T cells, but not CD4 T cells, in the induction of acute anemia during T. b. brucei infection.

## Discussion

Recently it was shown that enhanced erythrophagocytosis by activated liver neutrophils and monocytic cells, as well as spleen resident macrophages is responsible for the induction of acute anemia during *T*. *b*. *brucei* infection [19]. Here, we elucidate the mechanism behind this phenomenon. We show that upon *T*. *b*. *brucei* infection, NK, NKT and CD8 T cells rapidly produce IFNγ, which recruits neutrophils and monocyte-derived macrophages to liver and spleen, activating them to phagocytoze RBCs and consequently induce acute anemia.

Trypanosome infections are known to induce inflammation and inflammation-associated pathology [6,8–10,12–16]. In murine *T*. *b*. *brucei* infection this is characterized by an early type 1 cytokine storm and the occurrence of acute anemia [14,17]. Previous research has established an important role for IFNγ in macrophage priming and consequent type 1 cytokine production [2,3,6,20]. We show that liver and spleen NK and NKT cells are the early sources of IFNγ in *T*. *b*. *brucei* infection. While it is commonly known that these innate lymphoid cells are permanently in a ‘pre-primed state’ which allows them to respond rapidly to multiple infections [27,31–34], this is the first evidence that these cells play a role in the regulation of *T*. *b*. *brucei* infection-associated inflammation. Upon murine *T*. *b brucei* infection, NK cells can get activated in a non-selective way [28]. However, for example during MCMV infection, NK cells have been reported to react in an antigen-specific way [27,28]. During infection, NKT cells could get activated by parasite-derived glycolipid antigens such as the glycosylphosphatidylinositol (GPI) anchor of VSG [35], however recent evidence showed that innate stimuli such as IL-12 and toll like receptors (TLRs) are a major mean of NKT cell activation [29]. In addition, studies of CMV infection have shown that NKT cells activate and enhance NK cell responses [32]. The exact mechanism of NK and NKT cell activation during Trypanosome infection, and to what extent they play a role in host protection, is however the subject of a different study. By day six post infection CD4 and CD8 T cells get activated and accumulate in liver in spleen. This coincides with a shift in IFNγ producing cells. In the liver CD8 T cells seem to take over IFNγ production while in the spleen CD8 and CD4 T cells both become the principal IFNγ producing cells. Antigen-specific T cell activation during *T*. *b*. *brucei* infection has been extensively described [9,10,36], and a T cell-dependent antibody response is crucial for control of the first parasitemia peak [37,38]. Non-specific activation of CD8 T cells has also been reported during *T*. *b*. *brucei* infection via a trypanosome-derived molecule called TLTF, which supposedly acts directly on CD8 T cells to induce IFNγ production [22]. IFNγ production during the early stage of *T*. *b*. *brucei* infection is essential for recruitment of myeloid phagocytic cells to liver and spleen. Indeed, in the absence of IFNγ the myeloid cell composition of liver and spleen closely resembles that of a naïve C57BL/6 mouse. IFNγ has been previously implicated in recruitment of TNF- and iNOS-producing Tip-DCs to liver during *T*. *b*. *brucei* infection [39]. The phenotype of these TIP-DCs closely resembles that of the monocyte-derived macrophages described here. In addition, recruitment of both cell types is CCR2-dependent (S3C Fig), indicating that these are most likely the same cells. However, due to the expression of F4/80 and MerTK by these cells, we favor the terminology monocyte-derived macrophages. Using the newly developed pHrodo assay, we showed that IFNγ is not only needed for the recruitment of monocytes and neutrophils to spleen and liver, but is also necessary to activate these cells as well as the resident macrophages of the spleen. Indeed, in the absence of IFNγ, these cells displayed a reduced phagocytozing potential. It must be mentioned that not only the liver myeloid cell composition of infected IFNγR-/- mice resembles that of naïve mice, but also the erythrophagocytozing potential of each cell subset. Indeed, similar to naïve mice, resident macrophages or Kupffer cells are the only cells that display erythrophagocytozing potential [19]. In the spleen of IFNγR-/- mice the monocyte-derived macrophages are the major cells that display erythrophagocytosis. However, given the small size of this cell population, the contribution to acute anemia induction could be minor. Using the *in vitro* approach to monitor erythrophagocytosis we show that IFNγ can directly induce an enhanced erythrophagocytic potential. This activating potential of IFNγ is common, e.g. in Toxoplasma gondii infection IFNγ has been shown to act directly on macrophages to induced enhanced phagocytosis of RBC [25]. Of note, the results presented here do not prove that the direct effect of IFNγ on the myeloid cells is the only manner of enhancing erythrophagocytosis. Other parameters such as low-grade inflammation in the absence of IFNγ could also play a role.

In contrast to CD4 T cell depletion, depletion of both NK and NKT cells or CD8 T cells conferred protection against anemia. This coincided with reduced local and systemic IFNγ levels, confirming that these cells are the major IFNγ producers during early infection. The reduced anemic phenotype upon NK1.1 depletion could indicate that these cells are necessary for CD8 T cell activation in a non-specific way. Alternatively, it could be that a certain threshold level of IFNγ needs to be reached and that therefore concomitant IFNγ production by NK NKT and CD8 T cells is needed.

Previously we showed that during *T*. *b*. *brucei* infection an alteration of RBC membrane occurs, which coincided with an enhanced fragility and erythrophagocytosis by myeloid phagocytic cells of both naïve and infected animals [19]. Here we showed that RBCs from infected IFNγR-/- mice are equally fragile as RBCs from infected wild type mice, indicating that this process occurs independently of IFNγ. The altered RBC fragility in IFNγR-/- mice could still prime them for more rapid phagocytosis.

The data presented in this paper show that in the absence of IFNγ, mice are protected from infection-associated acute anemia. It is interesting to mention that IFNγ-/- mice die within 20 days of infection, in contrast to wild type mice, which die around day 35 post infection [21], which could indicate that the protection against acute anemia is of no clinical significance. In contrast, the enhanced RBC clearance early in infection could even be a protective mechanism as it could be a mean of the host to diminish iron availability, hereby ‘starving’ the parasite and impeding its growth. This could be an explanation for the higher parasitemia peak in IFNγR-/- mice compared to wild type mice [21], however it was also reported that there is no correlation between parasite load and anemia induction [17], arguing against correlations between anemia and parasitemia.

In conclusion, this work describes the mechanism behind the induction of acute anemia during *T*. *b*. *brucei* infection. IFNγ derived from NK, NKT and CD8 T cells is crucial for the recruitment and activation of myeloid phagocytic cells in liver and spleen and consequently for the induction of acute anemia. Whether this mechanism can be extrapolated to other trypanosome infections inducing acute anemia (such as *T*. *congolense* and *T*. *evansi*) remains to be investigated.

## Supporting Information

S1 Fig Myeloid cell gating strategy. A) First, CD45^+^ cells were selected based on a FSC-A/CD45 profile followed by gating on single cells (SSC-A/FSC-W profile). Then, CD11b^+^ cells were selected using an CD11b/FSC-A profile within the CD45^+^ population. Subsequently, neutrophils (CD11b^+^Ly6c^int^Ly6G^+^) were identified using a Ly6G/FSC-A profile and the remaining cells were used in an Ly6C versus MHC-II profile to identify monocytes (CD11b^+^Ly6c^high^Ly6G^-^MHC-II^-^), monocyte-derived macrophages (CD11b^+^Ly6c^high^Ly6G^-^MHC-II^+^), resident macrophages (CD11b^+^Ly6c^-^Ly6G^-^MHC-II^-^) and a Rest fraction (CD11b^+^Ly6c^-^Ly6G^-^MHC-II^-^). B) Surface marker expression on different myeloid cell subsets. Expression of F4/80, CCR2, Ly6B, MerTK and CD64 is displayed. C. Myeloid cell composition of liver in absolute cell numbers. D. Myeloid cell composition of spleen in absolute cell numbers. Values represent mean ± SD of 4 mice per group. *: p-value < 0.05 and if nothing is mentioned the differences were not significant.
(TIF)

S2 Fig Analysis of IFNγ producing cells in IFNγ reporter mice. A) First, CD45^+^ cells were selected based on a FSC-A/CD45 profile followed by gating on single cells (SSC-A/FSC-W profile). Then IFNγ+ cells were selected based on IFNγ versus FSC-A plot. Subsequently NK (TCRbeta- NK1.1+) and NKT (TCRbeta+ NK1.1+) cells were identified by plotting TCRbeta against NK1.1. TCRbeta+ NK1.1- cells were subsequently plotted on a CD8 versus CD4 graph. B) IFNγ in serum and spleen cell culture of naïve and day 5 infected GREAT IFNγ reporter mice.
(TIF)

S3 Fig Reconstitution confirmation. A) Confirmation of NK1.1 depletion in C57BL/6 mice day 6 post infection (pi). B) CD8 T cells were CFSE-labeled prior to adoptive transfer to CD8-/- mice. CFSE-labeled CD8 T cells were present in the spleen of reconstituted mice. C) Monocyte-derived macrophages depicted as a percentage of liver CD45+ cells in C56BL/6, IFNγR-/- and CCR2-/- mice. Values represent mean +/- 4 mice per group. A representative of two independent experiments is shown. ****: p-value < 0.0001 and if nothing is mentioned the differences were not significant.
(TIF)
