# Supplementary figures and images for: NK-, NKT- and CD8-Derived IFNγ Drives Myeloid Cell Activation and Erythrophagocytosis, Resulting in Trypanosomosis-Associated Acute Anemia
Source: PLoS Pathog. 2015 Jun 12;11(6):e1004964. doi: 10.1371/journal.ppat.1004964 (PMC4466398; doi:10.1371/journal.ppat.1004964)

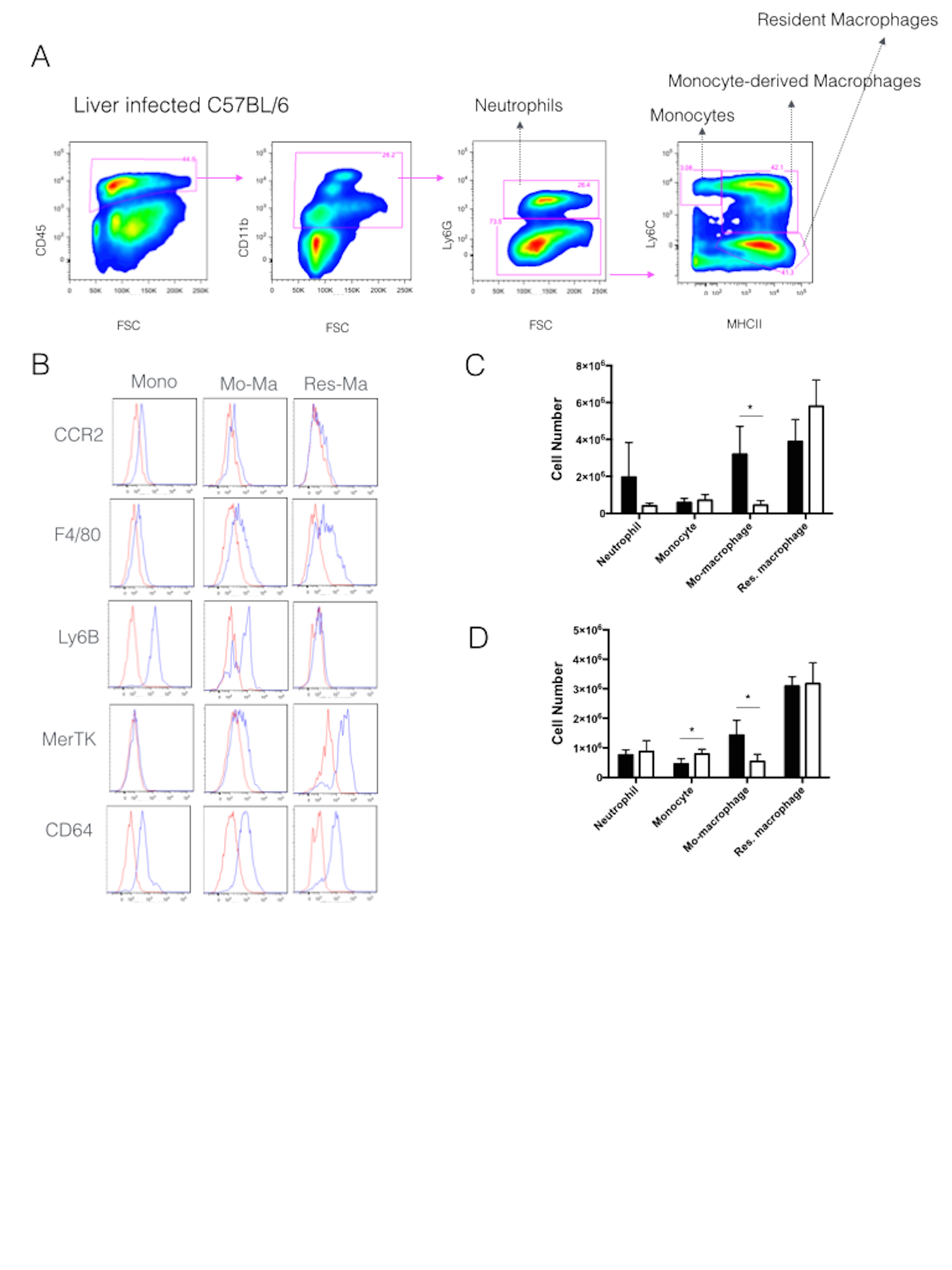

Supplement: S1 Fig — A) First, CD45+ cells were selected based on a FSC-A/CD45 profile followed by gating on single cells (SSC-A/FSC-W profile). Then, CD11b+ cells were selected using an CD11b/FSC-A profile within the CD45+ population. Subsequently, neutrophils (CD11b+Ly6cintLy6G+) were identified using a Ly6G/FSC-A profile and the remaining cells were used in an Ly6C versus MHC-II profile to identify monocytes (CD11b+Ly6chighLy6G-MHC-II-), monocyte-derived macrophages (CD11b+Ly6chighLy6G-MHC-II+), resident macrophages (CD11b+Ly6c-Ly6G-MHC-II-) and a Rest fraction (CD11b+Ly6c-Ly6G-MHC-II-). B) Surface marker expression on different myeloid cell subsets. Expression of F4/80, CCR2, Ly6B, MerTK and CD64 is displayed. C. Myeloid cell composition of liver in absolute cell numbers. D. Myeloid cell composition of spleen in absolute cell numbers. Values represent mean ± SD of 4 mice per group. *: p-value < 0.05 and if nothing is mentioned the differences were not significant. (TIF) [file ppat.1004964.s001.tif]

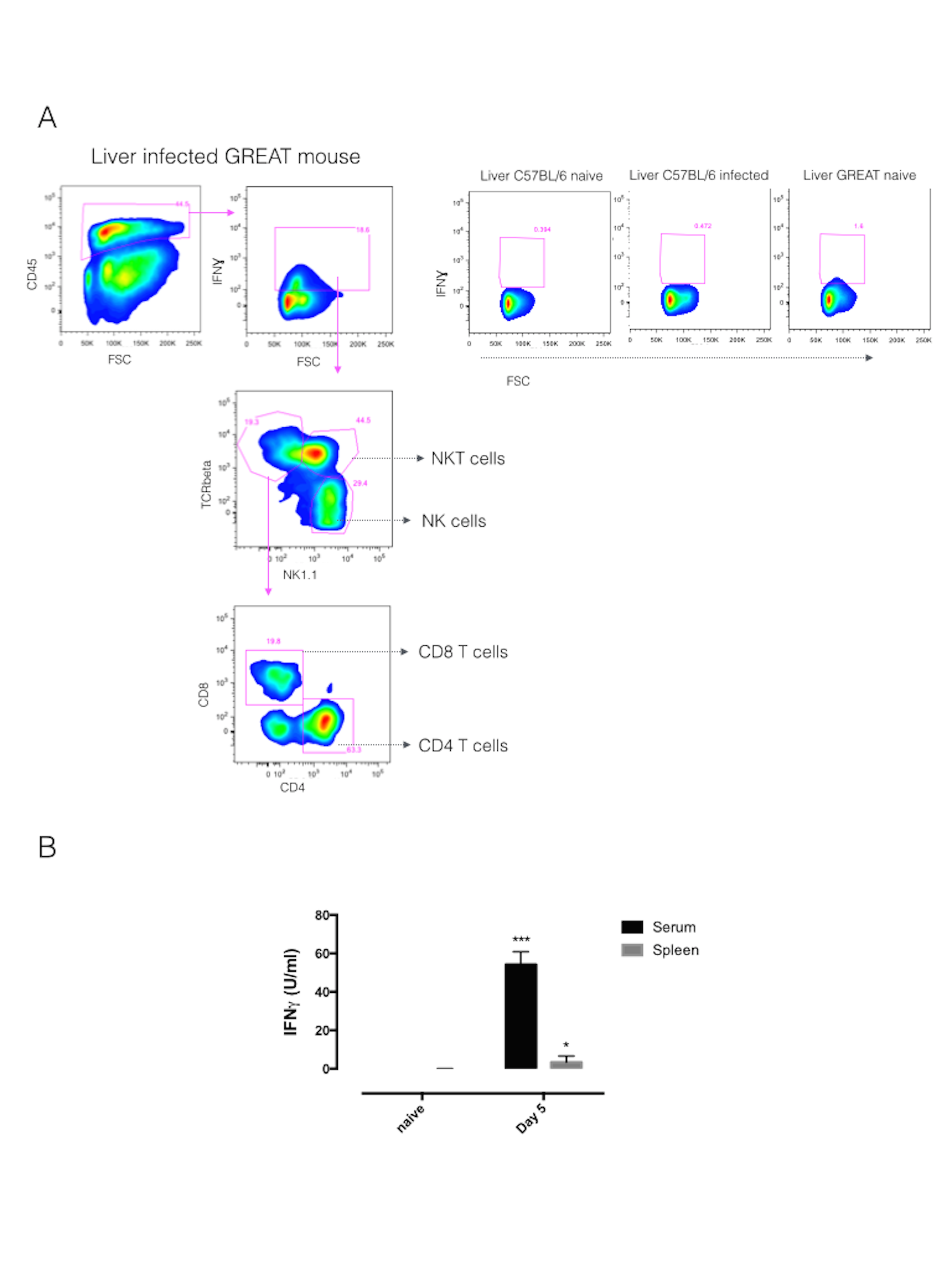

Supplement: S2 Fig — A) First, CD45+ cells were selected based on a FSC-A/CD45 profile followed by gating on single cells (SSC-A/FSC-W profile). Then IFNγ+ cells were selected based on IFNγ versus FSC-A plot. Subsequently NK (TCRbeta- NK1.1+) and NKT (TCRbeta+ NK1.1+) cells were identified by plotting TCRbeta against NK1.1. TCRbeta+ NK1.1- cells were subsequently plotted on a CD8 versus CD4 graph. B) IFNγ in serum and spleen cell culture of naïve and day 5 infected GREAT IFNγ reporter mice. (TIF) [file ppat.1004964.s002.tif]

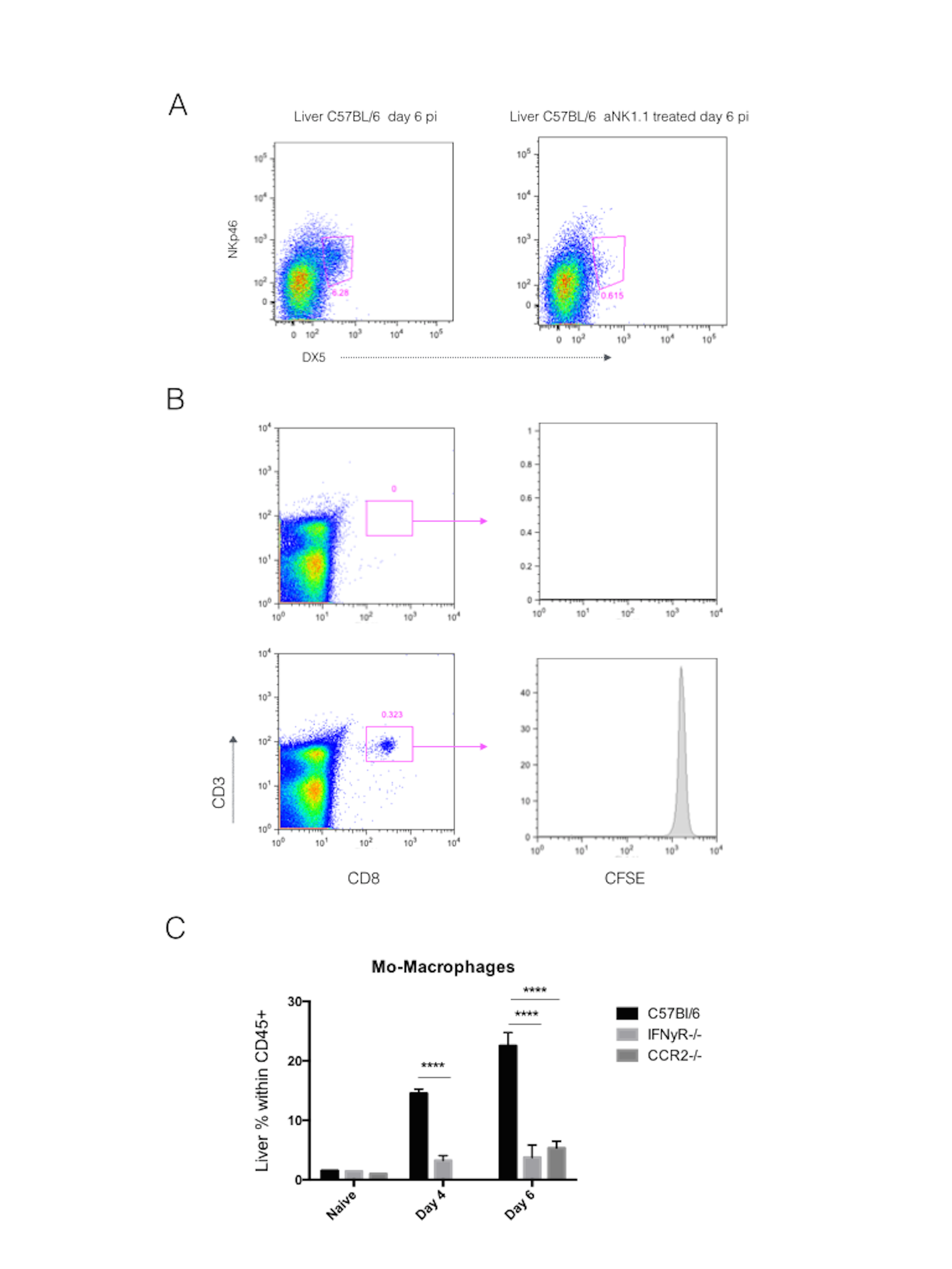

Supplement: S3 Fig — A) Confirmation of NK1.1 depletion in C57BL/6 mice day 6 post infection (pi). B) CD8 T cells were CFSE-labeled prior to adoptive transfer to CD8-/- mice. CFSE-labeled CD8 T cells were present in the spleen of reconstituted mice. C) Monocyte-derived macrophages depicted as a percentage of liver CD45+ cells in C56BL/6, IFNγR-/- and CCR2-/- mice. Values represent mean +/- 4 mice per group. A representative of two independent experiments is shown. ****: p-value < 0.0001 and if nothing is mentioned the differences were not significant. (TIF) [file ppat.1004964.s003.tif]
